# COVID-19 deaths in people with intellectual disability in the UK and Ireland: descriptive study

**DOI:** 10.1192/bjo.2020.102

**Published:** 2020-10-16

**Authors:** Bhathika Perera, Richard Laugharne, William Henley, Abigail Zabel, Kirsten Lamb, David Branford, Ken Courtanay, Regi Alexander, Kiran Purandare, Anusha Wijeratne, Vishwa Radhakrishnan, Eileen McNamara, Youshan Daureeawoo, Indermeet Sawhney, Mark Scheepers, Gordon Taylor, Rohit Shankar

**Affiliations:** Barnet Enfield and Haringey Mental Health Trust, UK; Cornwall Intellectual Disability and Epilepsy Research (CIDER) Centre, Cornwall Partnership Foundation NHS Trust, UK; University of Exeter Medical School, UK; Cornwall Intellectual Disability and Epilepsy Research (CIDER) Centre, Cornwall Partnership Foundation NHS Trust, UK; Royal College General Practitioners special interest group for learning disability, UK; UK; Barnet Enfield and Haringey Mental Health NHS Trust, UK; Hertfordshire Partnership University NHS Foundation Trust, UK; The Kingswood Centre, UK; The Kingswood Centre, UK; CNWL NHS Foundation Trust, UK; Barnet, Enfield and Haringey Mental Health trust, Barnet Learning Disability Team, UK; Barnet, Enfield and Haringey Mental Health trust, Barnet Learning Disability Team, UK; Hertfordshire Partnership University NHS Foundation Trust, UK; Gloucestershire Health and Care NHS Foundation Trust, UK; University of Exeter Medical School, UK; University of Exeter Medical School, UK

**Keywords:** COVID-19, intellectual disability deaths, premature mortality

## Abstract

**Background:**

Rapid spread of coronavirus disease 2019 (COVID-19) has affected people with intellectual disability disproportionately. Existing data does not provide enough information to understand factors associated with increased deaths in those with intellectual disability. Establishing who is at high risk is important in developing prevention strategies, given risk factors or comorbidities in people with intellectual disability may be different to those in the general population.

**Aims:**

To identify comorbidities, demographic and clinical factors of those individuals with intellectual disability who have died from COVID-19.

**Method:**

An observational descriptive case series looking at deaths because of COVID-19 in people with intellectual disability was conducted. Along with established risk factors observed in the general population, possible specific risk factors and comorbidities in people with intellectual disability for deaths related to COVID-19 were examined. Comparisons between mild and moderate-to-profound intellectual disability subcohorts were undertaken.

**Results:**

Data on 66 deaths in individuals with intellectual disability were analysed. This group was younger (mean age 64 years) compared with the age of death in the general population because of COVID-19. High rates of moderate-to-profound intellectual disability (*n* = 43), epilepsy (*n* = 29), mental illness (*n* = 29), dysphagia (*n* = 23), Down syndrome (*n* = 20) and dementia (*n* = 15) were observed.

**Conclusions:**

This is the first study exploring associations between possible risk factors and comorbidities found in COVID-19 deaths in people with intellectual disability. Our data provides insight into possible factors for deaths in people with intellectual disability. Some of the factors varied between the mild and moderate-to-profound intellectual disability groups. This highlights an urgent need for further systemic inquiry and study of the possible cumulative impact of these factors and comorbidities given the possibility of COVID-19 resurgence.

## Background

Studies have highlighted how coronavirus disease 2019 (COVID-19) continues to affect certain populations differently.^1^ Physical morbidities such as obesity, hypertension, cardiovascular disease and diabetes have been identified as high-risk factors and comorbidities, along with belonging to ethnic minorities in high- income countries, and also old age.^2,3^ This has led to developing risk stratification methods, so that preventative strategies such as shielding can be focused on individuals who belong to high-risk categories (https://www.coh-fit.com/about-the-project/).^3^

Over 500 people with an intellectual disability (also known as learning disability in UK health services) had died from COVID-19 in England by July 2020 (https://www.england.nhs.uk/publication/covid-19-deaths-of-patients-with-a-learning-disability-notified-to-leder/).^4^ Certain risk factors and comorbidities that are common in people with intellectual disability such as epilepsy and dysphagia are postulated to be possible reasons.^5^ People with intellectual disability are more likely to have severe physical illnesses and/or disabilities.^5^ A person with an intellectual disability may have numerous long-term conditions.^6^

Among people with intellectual disability, there is considerable over-representation of certain conditions such as epilepsy (22.5%) relative to the general population (0.6%).^7,8^ Epilepsy contributes to significantly higher premature mortality^9,10^ in people with intellectual disability. Pneumonia or aspiration pneumonia (to which dysphagia is a precursor) is considered a major risk factor for premature mortality for people with intellectual disability.^11^ Dysphagia was associated with 38% of all identified premature mortality in people with ID and combined with epilepsy was found in 18% of all premature ID deaths.^9^

Although it is recognised that genetic anomalies play a significant role in premature mortality in people with intellectual disability, they have been poorly researched. Common conditions such as Down syndrome are recognised to be overrepresented in the mortality figures. It is estimated that 13% of the premature mortality sample of Learning Disabilities Mortality Review (LeDeR) had Down syndrome.^9^ People with Down syndrome are highly vulnerable to dementia. Dementia is an associated factor for premature mortality with both epilepsy (11%) and dysphagia (13%).^9^

## Mental health problems and intellectual disability

Higher levels of mental health problems occur in people with intellectual disability.^12^ They independently contribute to 10–25 years earlier mortality. From LeDeR investigations it is known that 23% of all reported deaths were in individuals who had a mental illness diagnosis.^8^ In addition, there is recognition of the undue negative impact of psychotropic medication, such as antipsychotics, that negatively impact on premature mortality in people with intellectual disability.^8^

People with intellectual disability are more likely to require support from others including their families or paid carers in the community or in residential homes.^13,14^ Such models of care increase the social risk of transmission of infection from carers who often do shift work, possibly at multiple sites, as has occurred in care homes for people with dementia.^1^

There are no published reports looking at risk factors or comorbidities in people with intellectual disability. The risk of a resurgence of COVID-19 is high as only 6.5% of the population in the UK was reported to have antibodies to the virus in July 2020, far from reaching herd immunity.^15^ Consequently, physicians and psychiatrists caring for people with intellectual disability face the dilemma of identifying who is at higher risk from an already highly vulnerable population in order to take necessary measures to protect them from the infection and reduce mortality. Blanket isolation measures for a person with an intellectual disability can have an impact on their mental health given their routines and daily activities tend to be well structured and predictable. There is the added risk of inadvertent deprivation of liberty because of disproportionate social restrictive measures.^16^

## Aims

There is an urgent need to understand if general population risk factors and comorbidities apply synchronously to the intellectual disability population, and if there are added risk issues from known and common comorbid disorders and if so what their cumulative burden is.

## Method

### Study design

This is an observational descriptive study of people with intellectual disability who have died from COVID-19 to generate hypotheses on specific risk factors and comorbidities that may increase the risk of death from COVID-19 in this population.

Consultant psychiatrists and intellectual disability service leads in England and Ireland were contacted through various networks and asked to fill in a data sheet of possible risk factors and comorbidities in individuals with intellectual disability who had died because of COVID-19 from 1 March 2020 (chosen as a few days prior to the first death from COVID-19 reported in England) and 19 June 2020. A 2-week period from 8 June to 19 June was provided for data collection.

The numbers of deaths in people with intellectual disability was low. Therefore, data in this study was gained through an exponential non-discriminative snow-balling methodology^17^ in which psychiatrists and clinicians working in intellectual disability services collected data and provided further contacts who could provide more data on deaths in intellectual disability until the data collection deadline.

Demographic risk factors that are present in the general population and therefore may also be risk factors in people with intellectual disability were investigated. These included age, gender and ethnicity. We then identified health risk factors or comorbidities that have been shown to be risk factors in the general population for death from COVID-19.^2,3^ These included dementia, diabetes, hypertension, obesity, smoking status and asthma. Finally, a panel of experts in managing physical and mental health conditions in people with intellectual disability (consultant psychiatrists, a general practitioner, a pharmacist, academics in research methodology and statistics) hypothesised possible specific risk factors and comorbidities for people with intellectual disability based on available evidence. These were severity of intellectual disability, need for multiple carers, epilepsy, presence of dysphagia, genetic syndromes specifically Down syndrome, autism, attention-deficit hyperactivity disorder (ADHD), mental illness, challenging behaviour and prescriptions for antipsychotics.

The prevalence of individual risk factors or comorbidities, and their combination was examined in those who died. The severity of intellectual disability was divided into mild and moderate-to-profound intellectual disability.

### Rationale of dividing the group in to mild and moderate-to-profound intellectual disability

- Each of the three subgroups of ICD-10^18^ moderate (F71), severe (F72) and profound intellectual disability (F73) have a low prevalence (9% moderate intellectual disability, 4% severe intellectual disability, and about 2% profound) and together they would comprise 15% of the total intellectual disability population.^19^ Taken individually it would be difficult to achieve satisfactory power to deliver meaningful conclusions.
- Moderate, severe and profound intellectual disability is difficult to assess and classify, which causes significant issues with the accuracy of specific diagnosis of degree of intellectual disability.

### Ethics

Each site investigator submitted the project protocol and sample excel datasheet to their local health/social care organisations’ information and governance leads to obtain written permission to access health records of those who had died due to COVID-19 while their case was still open to the organisation. Each site confirmed that ethical clearance is not necessary. Data on deaths were anonymised at the site level before submitting data to the data coordinator for analysis. Posthumous investigations with anonymous data are exempted from undergoing formal ethical assessment.

Differences in the prevalence of risk factors or comorbidities, between the mild and moderate-to-profound intellectual disability subgroups was tested using Fisher's exact test with significance accepted at *P* ≤ 0.05. STrengthening the Reporting of OBservational studies in Epidemiology (The STROBE Checklist) was used to guide and report this observational study (see Supplementary data 1 available at https://doi.org/10.1192/bjo.2020.102).

## Results

During the 2 weeks of data collection, we received data on 82 COVID-19-related deaths in intellectual disability from learning disability services in England and Ireland. In total 20 clinicians from 18 sites contributed. Of these, two sites were excluded from data analysis as not enough information on risk factors or comorbidities were provided during the 2-week data collection window period. Therefore 66 deaths in England (*n* = 63) and Ireland (*n* = 3) were analysed in this paper.

**Fig. 1:**
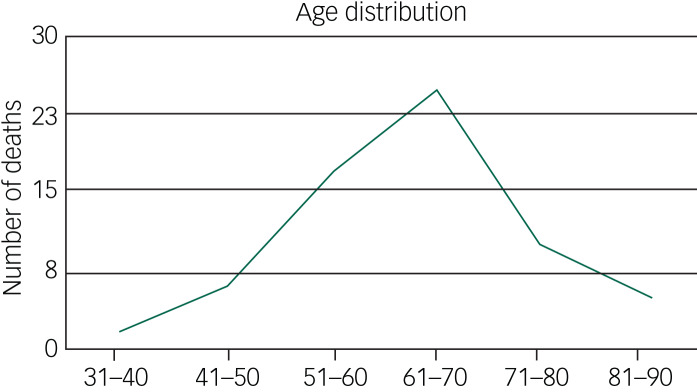
Age distribution of all COVID-19 deaths in people with intellectual disabilities.

Main findings are listed in Table 1. Data showed an age range of 31 to 88 with a median age of 64. Figure 1 shows the age distribution of all COVID-19 deaths. Of the deaths 39 were in men and 27 in women. Five patients were Black and minority ethnic.

**Table 1:** Prevalence of risk factors and comorbidities for COVID-19 deaths in people with intellectual disability (*n* = 66)

Factor	*n*	%
Age, years		
18 to <50	7	11
50 to <60	17	26
60 to <70	24	37
70 to <80	11	17
≥80	6	9
Gender		
Female	27	41
Male	39	59
Body mass index (BMI)		
Normal BMI	21	50
Overweight (25–30 BMI)	11	26
Obese (>30 BMI)	10	24
Missing	24	–
Smoking		
Never/former	54	96
Current	2	4
Missing	10	–
Ethnicity		
White	61	92
Asian	2	3
Black	2	3
Other	1	2
Severity of intellectual disability		
Mild	22	34
Moderate to profound	43	66
Unknown	1	–
Down syndrome		
No	46	70
Yes	20	30
Living setting		
Family home	11	17
Supported living	26	39
Residential care	21	32
Nursing care	8	12
Hypertension		
No	48	81
Yes	11	19
Missing	7	–
Dysphagia		
No	41	64
Yes	23	36
Missing	2	–
Diabetes		
No	55	86
Yes	9	14
Missing	2	–
Autism		
No	58	91
Yes	6	9
Missing	2	–
Attention-deficit hyperactivity disorder		
No	65	98
Yes	1	2
Epilepsy		
No	37	56
Yes	29	44
Asthma		
No	55	83
Yes	11	17
Dementia		
No	48	76
Yes	15	24
Missing	3	–
Severe mental illness		
No	55	83
Yes	11	17
Other mental illness		
No	47	72
Yes	18	28
Missing	1	–
Challenging behaviour		
No	43	65
Yes	23	35
Currently on antipsychotics		
No	46	71
Yes	19	29
Missing	1	–

In total, 54 of the 66 deceased never smoked. Data on body mass index (BMI) were missing for nearly a third of people. Of the remaining available data, 21 of the deaths were recorded in individuals within the normal BMI range and another 21 were categorised as either overweight or obese.

Regarding physical health conditions, the most common was a diagnosis of epilepsy (*n* = 29) followed by dysphagia (*n* = 23), dementia (*n* = 15), hypertension (*n* = 11), asthma (*n* = 11) and diabetes (*n* = 9).

Considering mental health parameters, 11 had a diagnosis of severe mental illness. In total, 18 were listed as having any other mental illnesses, with a total of 29 of the sample having some type of mental illness. Just over a third (*n* = 23) had presented with challenging behaviour. There were 19 who were on antipsychotic medications.

Approximately a third (*n* = 20) of the sample had Down syndrome. Six of the deaths of people with Down syndrome were in people with mild intellectual disability and 14 were in people with moderate-to-profound intellectual disability. The age range for deaths in Down syndrome was 44–67 and the median age was 58.

The number of deaths in people diagnosed with autism (*n* = 6) and ADHD (*n* = 1) were very low.

## Discussion

To our knowledge, this is the only case series examining probable risk factors and comorbidities in people with intellectual disability for COVID-19, with data collected systematically on deaths in people with intellectual disability because of COVID-19 in England and Ireland. The analysis of different demographic and physical and mental health data gives an insight into the possible factors associated with COVID-19 deaths in people with intellectual disability. Reports on increased mortality and a predicted second wave of COVID-19 highlight the importance of exploring specific factors and comorbidities that may put people with intellectual disability at greater risk without delay.

This discussion will focus on demographic and physical and mental health data identified in this study compared with the existing evidence base for deaths in people with intellectual disability prior to COVID-19.^9^

### Demographic data

Data for age-specific mortality from COVID-19 in the general population has shown that the age group of 90 years and over has the highest risk of death from COVID-19. Our case series shows that the median age group is 64, which is younger compared with a non-intellectual disability group.^3^ However, this needs to be read with caution given that average age expectation is lower in people with intellectual disability compared with a non-intellectual disability population.

### Physical and mental health considerations

It is well established that people with intellectual disability have more health problems compared with people without intellectual disability.^5^ Furthermore, the prevalence of certain physical health conditions is higher in people with moderate-to-profound intellectual disability compared with people with mild intellectual disability. Of particular interest is the subanalysis of the prevalence of epilepsy within the intellectual disability group with an increased prevalence of epilepsy occurring in mild intellectual disability (9/22 *v.* 20/43 in the moderate-to-profound intellectual disability group) but with no statistical difference between the two groups. People with mild intellectual disability have a 8–10% seizure comorbidity compared with 30–50% in the moderate-to-profound group.^7,8^ This generates a hypothesis that epilepsy may be an associated factor for death from COVID-19 in this population and particularly in the mild intellectual disability group.

A prevalence rate of 9.8% for hypertension is reported for people with intellectual disability;^20^ however, presence of hypertension in 11 out of 59 COVID-19 intellectual disability deaths suggests a higher prevalence in this smaller database. Of particular interest is that hypertension was a more significantly associated factor in people with mild intellectual disability than in those with moderate-to-profound intellectual disability (Table 2).

**Table 2:** Comparison of risk characteristics between individuals with mild and moderate-to-profound intellectual disability

Factor	Mild ID (*n* = 22)	Moderate to profound ID (*n* = 44)	*P*-value
Age > 70	32% (*n* = 7)	21% (*N* = 9)	0.38
Male	50% (*n* = 11)	65% (*n* = 28)	0.29
BAME	9% (*n* = 2)	7% (*n* = 3)	1.00
Obese	23% (*n* = 5)	12% (*N* = 5)	0.29
			
Hypertension	32% (*n* = 7)	9% (*n* = 4)	0.03
Diabetes	18% (*n* = 4)	12% (*n* = 5)	0.47
Dementia	27% (*n* = 6)	21% (*n* = 9)	0.76
Asthma	9% (*n* = 2)	21% (*n* = 9)	0.31
			
Autism	5% (*n* = 1)	12% (*n* = 5)	0.65
ADHD	0% (*n* = 0)	2% (*n* = 1)	1.00
Dysphagia	18% (*n* = 4)	44% (*n* = 20)	0.05
Epilepsy	41% (*n* = 9)	45% (*n* = 20)	0.79
Mental illness	41% (*n* = 9)	39% (*n* = 17)	1.00
			
Challenging behaviour	32% (*n* = 7)	37% (*n* = 16)	0.86
Currently on antipsychotics	27% (*n* = 6)	30% (*n* = 13)	0.50

There is no pre-COVID-19-specific mortality data for people with intellectual disability for hypertension, diabetes or asthma, which limits the ability to understand the importance of these health parameters found in the current data sample. Dysphagia has been recognised to be significantly associated with aspiration pneumonia and increased mortality in people with intellectual disability. Both dysphagia and associated mortality are significantly higher in people with moderate-to-profound intellectual disability.^21^ In our case series 19 out of 43 deaths in individuals with moderate-to-profound intellectual disability had a diagnosis of dysphagia compared with 4 out of 22 in the mild intellectual disability group.

Approximately a third (*n* = 20) of individuals who died had a diagnosis of Down syndrome. Prevalence of Down syndrome in intellectual disability is around 2.5%.^22^ The finding that 20 of the people with intellectual disability who died had Down syndrome is a cause for concern. This is also higher compared with LeDeR data,^9^ which reports approximately 16% of deaths in their cohort had Down syndrome. However, the median age of death of 58 found in this sample is similar to findings in the LeDer data. It can be assumed that the increased prevalence of respiratory conditions and other health conditions found in people with Down syndrome can predispose them to COVID-19 complications.

There is growing evidence that people with dementia are highly vulnerable to COVID-19.^3^ Dementia was over-represented in the deaths in this case series (15/48). We also considered ADHD and autism, the two most prevalent comorbid neurodevelopmental disorders among people with intellectual disability. They did not emerge as likely higher risk factors among people who died from COVID-19.

The high prevalence of mental illness in our sample compared with pre-COVID-19 intellectual disability premature mortality is an issue of concern. The presence of challenging behaviour as a risk factor in 23/66 of those who died, compared with a prevalence of 5–15% in the intellectual disability population^23^ needs to be noted. This could be because of sample bias as most data were collected by psychiatrists. No equivalent comparators are available from the general population or pre-COVID-19 intellectual disability deaths.

The use of antipsychotics in nearly a third of our sample compared with in 19% pre-COVID-19 intellectual disability deaths suggests the combined issue of mental health, challenging behaviour and antipsychotic use requires further closer scrutiny, especially when confounded by epilepsy and possibly its treatment. However, it is again worth noting that data were collected by psychiatrists who have access to data for people with intellectual disability and mental health issues.

Of all those who died, 55 out of 66 lived in supported living, residential or nursing homes. This leads to a hypothesis as to whether exposure to multiple carers is associated with a high risk of dying from COVID-19. This may not be unexpected given the rates of deaths in care homes caring for people with dementia because of COVID-19 in the UK. Establishing this as a risk factor along with other risk factors would help to identify who is at high risk of dying from COVID-19, and help to establish specific measures that could be implemented to protect those at high risk who are cared for by multiple carers rather than a blanket restriction on everyone living in a care setting.

### Limitations and strengths

As a case series collated through a snow-balling methodology we do not claim it is a systemically representative sample. It is not possible to determine the actual population pattern of distribution. The use of a peer network can lead to oversampling bias. Psychiatrists were reached through different networks, so it is difficult to know the number of people reached. The use of psychiatrists as the data collection medium may have caused high prevalence figures for mental illness and antipsychotic usage. Further, it is not possible to determine the sampling error and make statistical inferences from the sample to the ID population because of the absence of a random selection of samples. In particular there is a risk of type 2 error when comparing the conditions between the mild and moderate-to-profound groups. Therefore it can only generate hypotheses that need further testing through more systematic research methodology.

### Implications for research

This case series has generated the hypotheses that people with intellectual disability may be more likely to die from COVID-19 infection if there is the presence of a single or a combination of factors or comorbidities, These include moderate-to-profound intellectual disability, epilepsy, living setting, dysphagia, dementia, mental illness and Down syndrome. There also appears to be certain possible associated-factor differences between people with mild intellectual disability compared with those with moderate-to-profound intellectual disability. These hypotheses need to be tested by more rigorous methodologies. Some factors may confound others and should be examined further.

Future studies assessing risk factors and comorbidities would need to consider a system that can collect data on deaths in intellectual disability because of COVID-19 in a systematic way and for comparison with a larger group of people with intellectual disability (of similar nature and degree) without COVID-19 complications. It also needs to be explored if people with intellectual disability in supported environments are more prone to infection and death, and if so, what features of a care establishment that increase risks.

### Implications for clinical and social practice

Future studies should focus on developing risk scores. Risk scores will help to provide an understanding of the cumulative burden of certain key conditions and more specific public health messages for people with intellectual disability. Guidance is needed on the stratification of risk for people with intellectual disability so that the appropriate level of shielding can be given to individuals. This may be the start of that process. Even though our data suggests that people with intellectual disability with Down syndrome, dementia, epilepsy, moderate-to-profound intellectual disability, mental illness and dysphagia may be vulnerable to death from COVID-19 infection, it is too early to draw conclusions. The finding that 75% of those who died had at least two risk factors or comorbidities could be used to actively identify those at great risk in an already vulnerable population.

### Implications for policy

The protection of individuals with intellectual disability in supported environments needs to be prioritised in any outbreak of COVID-19. There are clearly lessons to be learned from care homes for elderly people that are likely to also apply to care facilities for people with intellectual disability, and policies will need to be developed to protect people with intellectual disability. The moral welfare of any society can be judged by the care of those most vulnerable.

## Data Availability

The data that support the findings of this study are available from the corresponding author upon reasonable request.

## Supplementary material

For supplementary material accompanying this paper visit http://dx.doi.org/10.1192/bjo.2020.102. click here to view supplementary material
